# The Mu Opioid Receptor Promotes Opioid and Growth Factor-Induced Proliferation, Migration and Epithelial Mesenchymal Transition (EMT) in Human Lung Cancer

**DOI:** 10.1371/journal.pone.0091577

**Published:** 2014-03-24

**Authors:** Frances E. Lennon, Tamara Mirzapoiazova, Bolot Mambetsariev, Valeriy A. Poroyko, Ravi Salgia, Jonathan Moss, Patrick A. Singleton

**Affiliations:** 1 Section of Pulmonary and Critical Care, Department of Medicine, Pritzker School of Medicine, The University of Chicago, Chicago, Illinois, United States of America; 2 Department of Surgery, Pritzker School of Medicine, The University of Chicago, Chicago, Illinois, United States of America; 3 Section of Hematology/Oncology, Department of Medicine, Pritzker School of Medicine, The University of Chicago, Chicago, Illinois, United States of America; 4 Department of Anesthesia and Critical Care, Pritzker School of Medicine, The University of Chicago, Chicago, Illinois, United States of America; Ghent University, Belgium

## Abstract

Recent epidemiologic studies implying differences in cancer recurrence based on anesthetic regimens raise the possibility that the mu opioid receptor (MOR) can influence cancer progression. Based on our previous observations that overexpression of MOR in human non-small cell lung cancer (NSCLC) cells increased tumor growth and metastasis, this study examined whether MOR regulates growth factor receptor signaling and epithelial mesenchymal transition (EMT) in human NSCLC cells. We utilized specific siRNA, shRNA, chemical inhibitors and overexpression vectors in human H358 NSCLC cells that were either untreated or treated with various concentrations of DAMGO, morphine, fentanyl, EGF or IGF. Cell function assays, immunoblot and immunoprecipitation assays were then performed. Our results indicate MOR regulates opioid and growth factor-induced EGF receptor signaling (Src, Gab-1, PI3K, Akt and STAT3 activation) which is crucial for consequent human NSCLC cell proliferation and migration. In addition, human NSCLC cells treated with opioids, growth factors or MOR overexpression exhibited an increase in snail, slug and vimentin and decrease ZO-1 and claudin-1 protein levels, results consistent with an EMT phenotype. Further, these effects were reversed with silencing (shRNA) or chemical inhibition of MOR, Src, Gab-1, PI3K, Akt and STAT3 (p<0.05). Our data suggest a possible direct effect of MOR on opioid and growth factor-signaling and consequent proliferation, migration and EMT transition during lung cancer progression. Such an effect provides a plausible explanation for the epidemiologic findings.

## Introduction

The role of anesthesia and analgesia in the recurrence and metastatic rate of malignancies has recently received considerable attention [1], [2], [3], [4]. Retrospective studies have demonstrated a diminished incidence of cancer recurrence following regional anesthesia with lower doses of opioids following surgery for breast, prostate, colon cancer and melanoma, although other studies have failed to detect significant differences [5], [6], [7], [8]. Some hypotheses to explain these differences in recurrence rates include immune suppressive effects and direct effects on tumor cell growth [9], [10], [11]. Our research has focused on the mu opioid receptor (MOR) and its role in directly regulating cellular changes leading to tumor growth and metastasis [4], [12], [13].

Effective therapeutic strategies for lung cancer, the leading cause of cancer-associated mortality worldwide, are extremely limited exemplifying the need for early diagnosis and novel therapeutic interventions [14], [15]. We have previously reported that the MOR is upregulated in several types of human non-small cell lung cancer (NSCLC) [12]. Further, we have shown that overexpression of MOR in human NSCLC increases primary tumor growth and metastasis in xenograft models [13]. However, the exact cellular changes regulated by MOR in NSCLC are incompletely defined [4]. For cancer cells to grow and metastasize, there needs to be a loss of cell-cell adhesion (characterized by a reduction of epithelial cell adhesion proteins including the tight junction proteins, ZO-1 and claudin-1) followed by acquisition of mesenchymal characteristics including a loss of baso-apical polarization, cytoskeletal remodeling and increased cell motility (characterized by increases in specific cytoskeletal proteins (i.e. vimentin) and transcription factors (i.e. Slug and Snail) [16], [17], [18], [19]. This orchestrated oncogenic process is referred to as epithelial mesenchymal transition (EMT) [16], [17], [18], [19], [20], [21], [22].

Growth factor receptors, including the epidermal growth factor receptor (EGFR), are often overexpressed and/or mutated in NSCLC and regulate oncogenic processes including tumor cell proliferation, migration and EMT transition [23], [24], [25], [26], [27]. Several therapies targeting the EGFR in NSCLC exist including tyrosine kinase inhibitors (gefitinib, erlotinib) and monoclonal antibodies (cetuximab)[28], [29], [30], [31]. However, the overall survival rate for NSCLC remains low [32], [33], [34]. Recently, Fujioka et al., have demonstrated that morphine can stimulate EGFR signaling pathways including the serine/threonine kinases Akt and MAP kinase in NSCLC suggesting a role for MOR inhibition as a potential therapeutic strategy for NSCLC [35].

Based on the recent interest of the effects of anesthesia and analgesia regimens on the recurrence and metastatic potential of various cancers [1], [2], [3], [4], our previous published data indicating the MOR is upregulated in lung tissue from patients with NSCLC [12], overexpression of MOR promotes tumor growth and metastasis in human NSCLC xenograft models [13] as well as data from Fujioka et al., demonstrating MOR regulation of EGF-induced signaling events in NSCLC [35], this study investigated the functional effects of MOR in the fundamental oncogenic processes of opioid and growth factor-induced human lung cell migration, proliferation and epithelial mesenchymal transition (EMT)[16], [17], [18], [19], [20]. Since there is currently very little information on opioid and/or MOR regulation of EMT and the molecular mechanisms integrating cancer cell proliferation, migration and EMT, this study investigated the detailed molecular mechanisms for these events which can have potential clinical utility.

## Methods

### Cell Culture and Reagents

The human NSCLC cell H358 was obtained from ATCC (Walkersville, MD) and cultured in Roswell Park Memorial Institute complete medium (Cambrex, East Rutherford, NJ) at 37°C in a humidified atmosphere of 5% CO2, 95% air, with passages 6–10 used for experimentation. Unless otherwise specified, reagents were obtained from Sigma (St. Louis, MO). Reagents for SDS-PAGE electrophoresis were purchased from Bio-Rad (Richmond, CA) and Immobilon-P transfer membrane was purchased from Millipore (Millipore Corp., Bedford, MA). Rabbit anti-MOR antibody was purchased from GeneTex (San Antonio, TX). Rabbit anti-EGFR, rabbit anti-phosphotyrosine-EGFR (pY^845^, pY^992^, pY^1045^, pY^1068^), rabbit anti-Grb-2, rabbit anti-Gab-1, rabbit anti-phosphotyrosine-Gab-1 (pY^307^, pY^627^), rabbit anti-Src, rabbit anti-phosphotyrosine-Src (pY^416^), rabbit anti-p85 PI3 kinase, rabbit anti-p55 PI3 kinase, rabbit anti-phosphotyrosine-p85/p55 PI3 kinase (pY^458^, pY^199^), rabbit anti-STAT3, rabbit anti-phosphotyrosine-STAT3 (pY^705^), rabbit anti-vimentin, rabbit anti-ZO-1, rabbit anti-claudin-1, rabbit anti-Snail and rabbit anti-Slug antibodies were purchased from Cell Signaling Technologies (Danvers, MA). Mouse anti-β-actin antibody was purchased from Sigma (St. Louis, MO). Secondary horseradish peroxidase-labeled antibodies were purchased from Amersham Biosciences (Piscataway, NJ). N-methylnaltrexone bromide or methylnaltrexone was purchased from Mallinckrodt Specialty Chemicals (Phillipsburg, NJ). The PI3 kinase inhibitor LY294002, Akt Inhibitor X, the Src family kinase inhibitor PP2 and the STAT3 inhibitor Stattic were purchased from EMD Biosciences (Billerica, MA).

### Immunoblotting

Immunoblotting was performed as we have previously described. Cellular materials from treated or untreated human NSCLC cells were incubated with lysis buffer (50 mM HEPES (pH 7.5), 150 mM NaCl, 20 mM MgCl_2_, 1% Triton X-100, 0.1% SDS, 0.4 mM Na_3_VO_4_, 40 mM NaF, 50 µM okadaic acid, 0.2 mM phenylmethylsulfonyl fluoride, 1∶250 dilution of Calbiochem protease inhibitor mixture 3). The samples were then run on SDS-PAGE in 4–15% polyacrylamide gels, transferred onto Immobilon™ membranes, and developed with specific primary and secondary antibodies. Visualization of immunoreactive bands was achieved using enhanced chemiluminescence (Amersham Biosciences, Piscataway, NJ). In some instances, immunoreactive bands were quantitated using computer-assisted densitometry.

### Small Interfering RNA Transfection in Human NSCLC Cells

Stable Control and either MOR, Gab-1, or Src siRNA (Santa Cruz Biotechnology, Santa Cruz, CA) were transfected into H358 cells as we have previously described [12]. Cells (∼40% confluent) were serum-starved for 1 hour followed by incubated with siRNA for 6 hours in serum-free media. Serum-containing media was then added (10% serum final concentration) for 42 hours. Inhibition of protein expression was confirmed by immunoblot analysis with specific antibodies.

### Stable Control and MOR Small Hairpin RNA Transfection in Human NSCLC Cells

Stable Control and MOR shRNA (Santa Cruz Biotechnology, Santa Cruz, CA) were stably transfected into H358 cells as we have previously described [12]. Cells (∼40% confluent) were serum-starved for 1 hour followed by incubated with shRNA for 6 hours in serum-free media. Serum-containing media was then added (10% serum final concentration) for 42 hours and puromycin selection reagent was added. Inhibition of protein expression was confirmed by immunoblot analysis with anti-MOR antibody (GeneTex, San Antonio, TX).

### Stable Vector Control and MOR1 Overexpression in Human NSCLC Cells

Myc-DDK-tagged ORF clone of Homo sapiens opioid receptor, mu 1 (OPRM1), transcript variant MOR-1 (OriGene Technologies Inc, MD) was amplified using Platinum Taq DNA polymerase high fidelity enzyme (Invitrogen, CA) and subsequently cloned into a pCR8/GW/Topo entry vector (Invitrogen, CA) according to manufacturer's instructions. Plasmid DNA was extracted from selected clones by QIAquick Plasmid Mini kit (Qiagen, CA). ORF integrity and fragment orientation were confirmed by sequencing. The MOR1-Myc fusion product was then transferred to pcDNA3.2/v5 DEST vector (Invitrogen, CA) by LR reaction. The resulting construct (pcDNA3.2-MOR1-Myc) was transfected into H358 cells using FuGENE HD™ as the transfection reagent (Roche Applied Sciences) according to the protocol provided by Roche as we have previously described. Cells (∼40% confluent) were serum-starved for 1 hour followed by incubation with pcDNA3.2-MOR1-Myc for 6 hours in serum-free media. Serum-containing media was then added (10% serum final concentration) for 42 hours and neomycin selection reagent was added. Overexpression was confirmed by immunoblot analysis with anti-MOR antibody (GeneTex, San Antonio, TX).

### Human NSCLC Cell Proliferation Assay

Measurement of *in vitro* NSCLC cell growth was performed as we have previously described. Control or siRNA pretreated H358 cells (5×10^3^ cells/well) were incubated with 0.2 ml of serum-free media containing either vehicle (control), methylnaltrexone (MNTX, 100 nM), the PI3 kinase inhibitor LY294002 (10 uM), Akt Inhibitor X (5 uM), the Src family kinase inhibitor PP2 (100 nM) or the STAT3 inhibitor Stattic (10 uM) for 72 h at 37°C in 5%CO2/95% air in 96-well culture plates. The *in vitro* cell proliferation assay was analyzed by measuring increases in cell number using the CellTiter96™ MTS assay (Promega, Madison, WI) and read at 492 nm. Each assay was set up in triplicate and repeated at least five times.

### Human NSCLC Cell Migration Assay

Measurement of *in vitro* NSCLC cell migration was performed as we have previously described. Twenty-four transwell units with 8 µM pore size (Millipore, Billerica, MA) were used for monitoring *in vitro* cell migration as we have previously described [12]. Control or siRNA pretreated H358 cells (5×10^3^ cells/well) were incubated with 0.2 ml of serum-free media containing either vehicle (control), methylnaltrexone (MNTX, 100 nM), the PI3 kinase inhibitor LY294002 (10 uM), Akt Inhibitor X (5 uM), the Src family kinase inhibitor PP2 (100 nM) or the STAT3 inhibitor Stattic (10 uM) were plated on the upper chamber and media with serum was added to the lower chamber. Cells were allowed to migrate through the pores for 18 hours. Cells from the upper and lower chamber were quantitated using the CellTiter96™ MTS assay (Promega, San Luis Obispo, CA) and read at 492 nm. % migration was defined as the # of cells in the lower chamber divided by the number of cells in both the upper and lower chamber. Each assay was set up in triplicate and repeated at least five times.

### Statistical Analysis

Results are expressed as mean ± standard deviation of three independent experiments. For data analysis, experimental samples were compared to controls by unpaired Student's t-test. For multiple-group comparisons, a one-way variance analysis (ANOVA) and post hoc multiple comparisons tests were used. Differences between groups were considered statistically significant when *P* value was less than 0.05. All statistical analyses were performed using the GraphPad Prism program (GraphPad Software Inc., USA).

## Results

**Figure 1:**
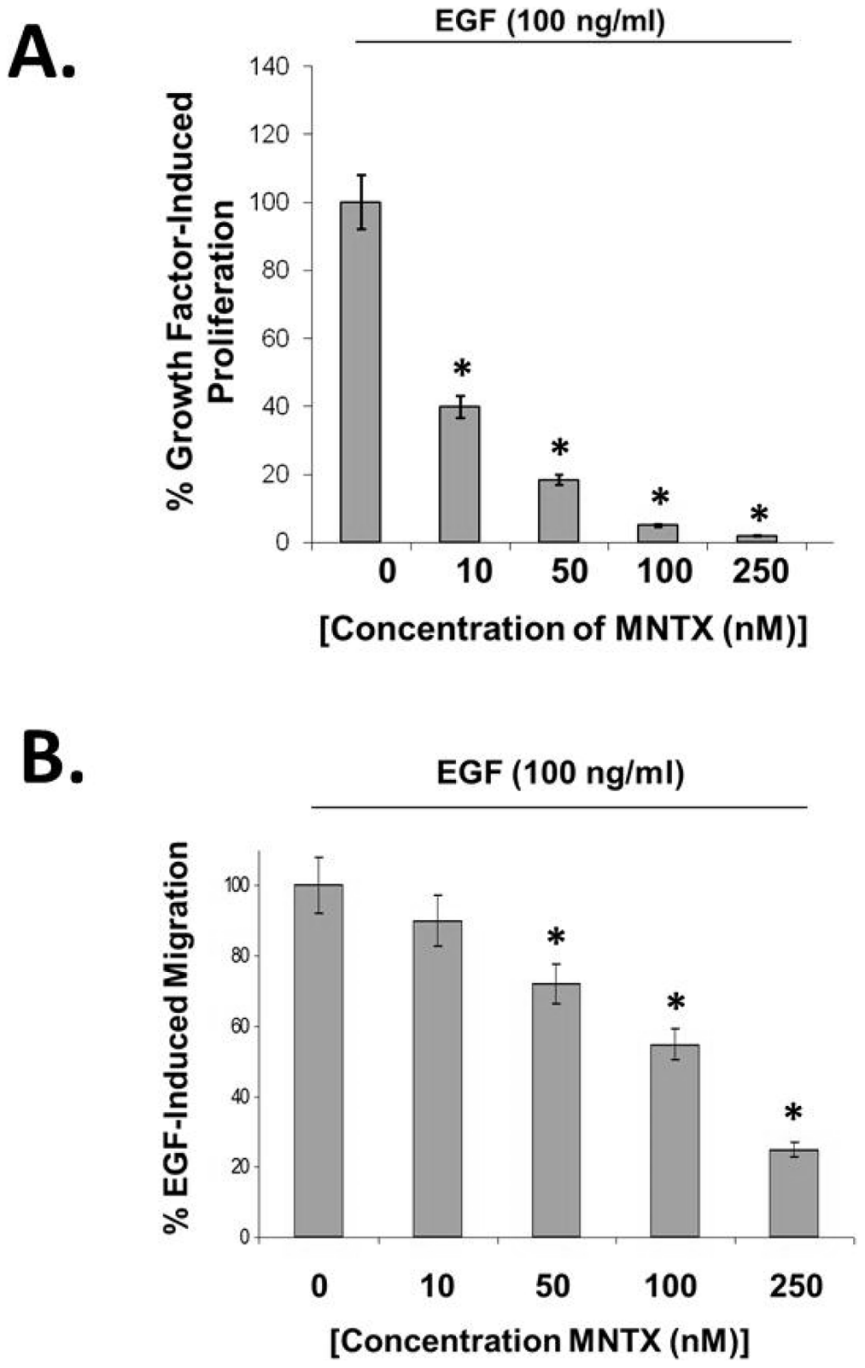
The peripheral mu opioid receptor antagonist, methylnaltrexone (MNTX), inhibits epidermal growth factor (EGF)-induced proliferation and migration of human lung cancer cells in a dose-dependent manner. **Panel A**: Human H358 non-small cell lung cancer (NSCLC) cells were analyzed for methylnaltrexone (MNTX) inhibition of EGF-mediated proliferation using a MTS proliferation assay. Cells were growth in the presence of 100 ng/ml EGF and/or 0–250 nM MNTX for 72 hours. MNTX There is a statistically significant difference (p<0.05, indicated by an asterisks) between control and MNTX (10, 50, 100, 250 nM) treatment with n = 3 per condition and error bars = standard deviation. See the Methods section for experimental details. **Panel B**: Human H358 non-small cell lung cancer (NSCLC) cells were analyzed for methylnaltrexone (MNTX) inhibition of EGF-mediated migration using a transwell assay (8 uM pore size). Cells were allowed to migrate in the presence of 100 ng/ml EGF and/or 0–250 nM MNTX for 18 hours. There is a statistically significant difference (p<0.05, indicated by an asterisks) between control and MNTX (50, 100, 250 nM) treatment with n = 3 per condition and error bars = standard deviation. See the Methods section for experimental details.

**Figure 2:**
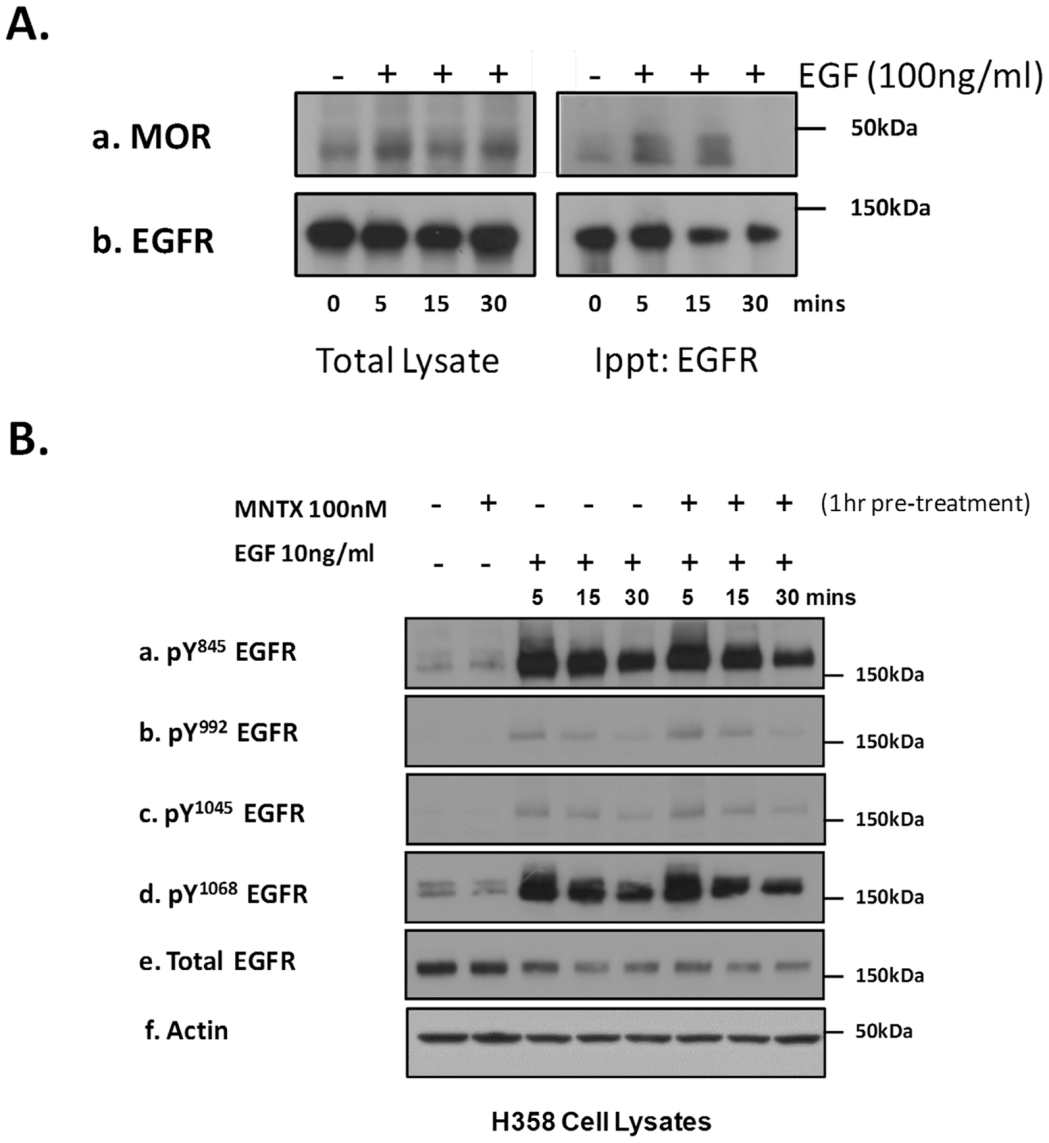
The mu opioid receptor (MOR) is recruited to the EGF receptor with EGF stimulation but does not regulate EGF receptor phosphorylation. **Panel A**: Human H358 non-small cell lung cancer (NSCLC) cells were treated with no (control) or 100 ng/ml EGF for 5, 15, or 30 minutes. Cell lysates were obtained and immunoprecipitated with anti-EGFR antibody. Immunoblots were performed on total cell lysates (left) and immunoprecipitated material (right) using anti-MOR (a) and anti-EGFR (b) antibodies. The mu opioid receptor is recruited to the EGFR with EGF stimulation. **Panel B**: Human H358 non-small cell lung cancer (NSCLC) cells were either untreated (control) or treated with 100 nM MNTX alone, 10 ng/ml EGF for 5, 15 or 30 minutes, or 100 nM MNTX and 10 ng/ml EGF for 5, 15, or 30 minutes. Cell lysates were obtained and immunoblotted using anti-pY^845^ EGFR (a), anti-pY^992^ EGFR (b), anti-pY^1045^ EGFR (c), anti-pY^1068^ EGFR (d), anti-EGFR (e) and anti-actin (e) antibodies. MNTX does not inhibit EGF-induced EGFR tyrosine phosphorylation.

Our results in Figure 1 indicate that inhibiting MOR with the peripheral MOR antagonist MNTX attenuates EGF-induced proliferation (Figure 1-A) and migration (Figure 1-B) of human H358 NSCLC cells in a dose-dependent manner. These data suggest a link between MOR and the EGFR in H358 cells. To mechanistically evaluate the role of MOR on EGF-induced EGFR dynamics, we treated H358 cells with EGF at various times and immunoprecipitated the EGFR to determine potential MOR association. Figure 2-A demonstrates that EGF induces a complex formation between the EGFR and MOR which peaks at 5 to 15 minutes after EFG challenge. Based on our results that a MOR/EGFR complex can occur with EGF stimulation of H358 cells, we next examined whether MOR can regulate EGFR phosphorylation. Utilizing a panel of anti-phospho-EGFR antibodies, Figure 2-B demonstrates that pretreatment of H358 human NSCLC cells with the peripheral MOR antagonist MNTX failed to attenuate EGF-induced EGFR tyrosine phosphorylation.

**Figure 3:**
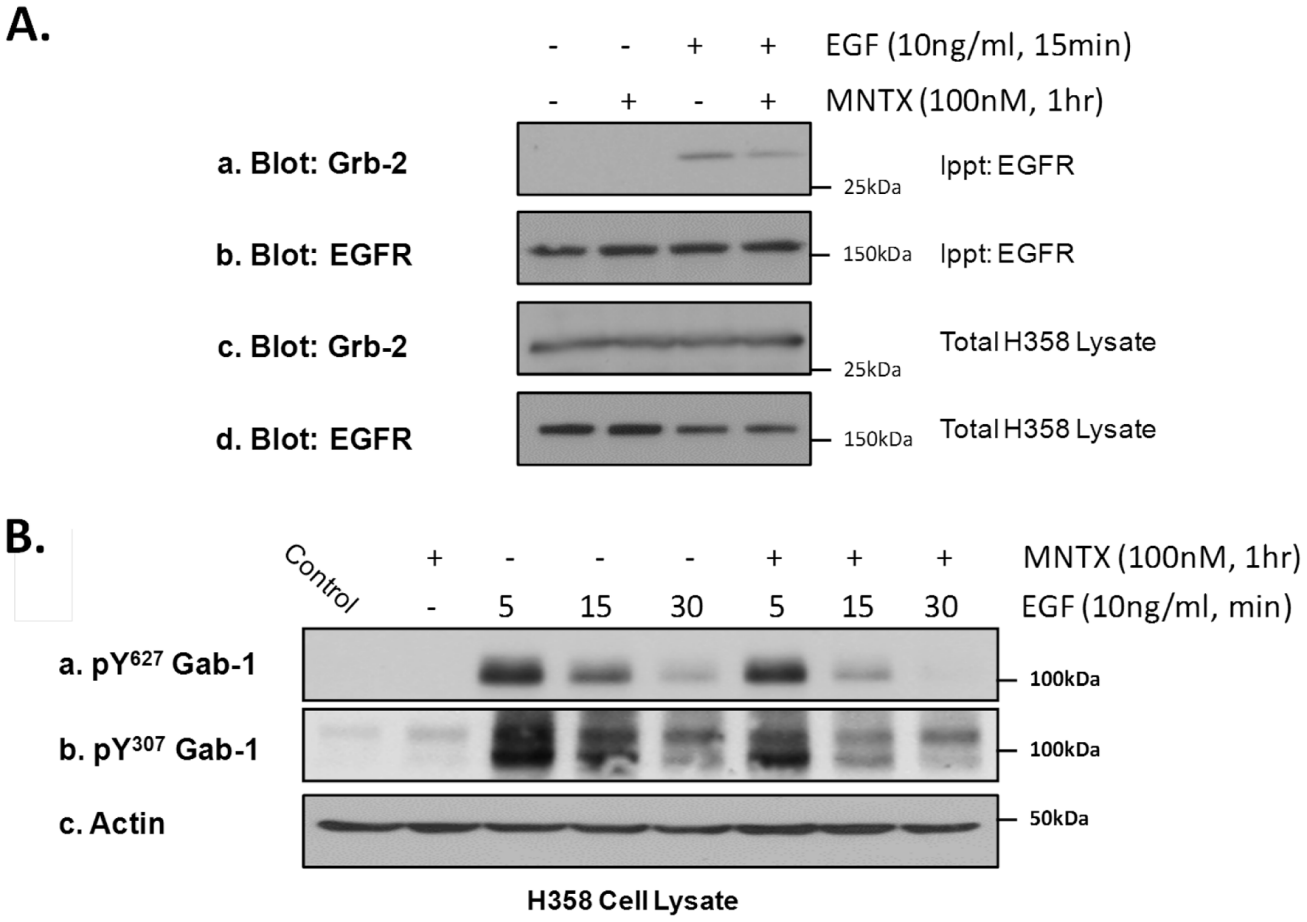
The peripheral mu opioid receptor antagonist, methylnaltrexone (MNTX), inhibits EGF-induced recruitment/activation of the linker protein, Grb-2, and the scaffolding protein, Gab-1, in human lung cancer cells. **Panel A**: Human H358 non-small cell lung cancer (NSCLC) cells were either untreated (control) or treated with 100 nM MNTX (1 hour pre-incubation), 10 ng/ml EGF for 15 minutes, or 100 nM MNTX and 10 ng/ml EGF. Cell lysates were obtained and immunoprecipitated with anti-EGFR antibody. Immunoblots were performed on total cell lysates and immunoprecipitated material using anti-Grb-2 (a,c) and anti-EGFR (b,d) antibody. MNTX inhibits EGF-induced recruitment of Grb-2 to the EGFR. **Panel B**: Human H358 non-small cell lung cancer (NSCLC) cells were either untreated (control) or treated with 100 nM MNTX alone, 10 ng/ml EGF for 5, 15 or 30 minutes, or 100 nM MNTX and 10 ng/ml EGF for 5, 15, or 30 minutes. Cell lysates were obtained and immunoblotted using anti-pY^627^ Gab-1 (a), anti-pY^307^ Gab-1 (b) and anti-actin (c) antibodies. MNTX attenuates EGF-induced Gab-1 tyrosine phosphorylation.

We next examined whether MOR can regulate EGFR downstream signaling molecules. We first examined the adaptor protein, Growth factor receptor-bound protein 2 (Grb-2), which contains an SH2 domain and two SH3 domains and can directly bind the EGFR [36]. The results of Figure 3-A demonstrate, using immunoprecipitation of EGFR and immunoblotting with anti-Grb-2 antibody, that EGF induces EGFR/Grb-2 complex formation which is attenuated by pretreatment of H358 cells with MNTX. Recruitment of Grb-2 to the EGFR is important for plasma membrane recruitment and consequent tyrosine phosphorylation of the scaffolding protein, Grb2-associated-binding protein 1 (Gab-1) [37]. Figure 3-B indicates that EGF stimulation of H358 cells induces tyrosine phosphorylation of Gab-1 (Tyr307 and Tyr627) which peaks at ∼5 minutes and is attenuated by MOR inhibition with MNTX.

**Figure 4:**
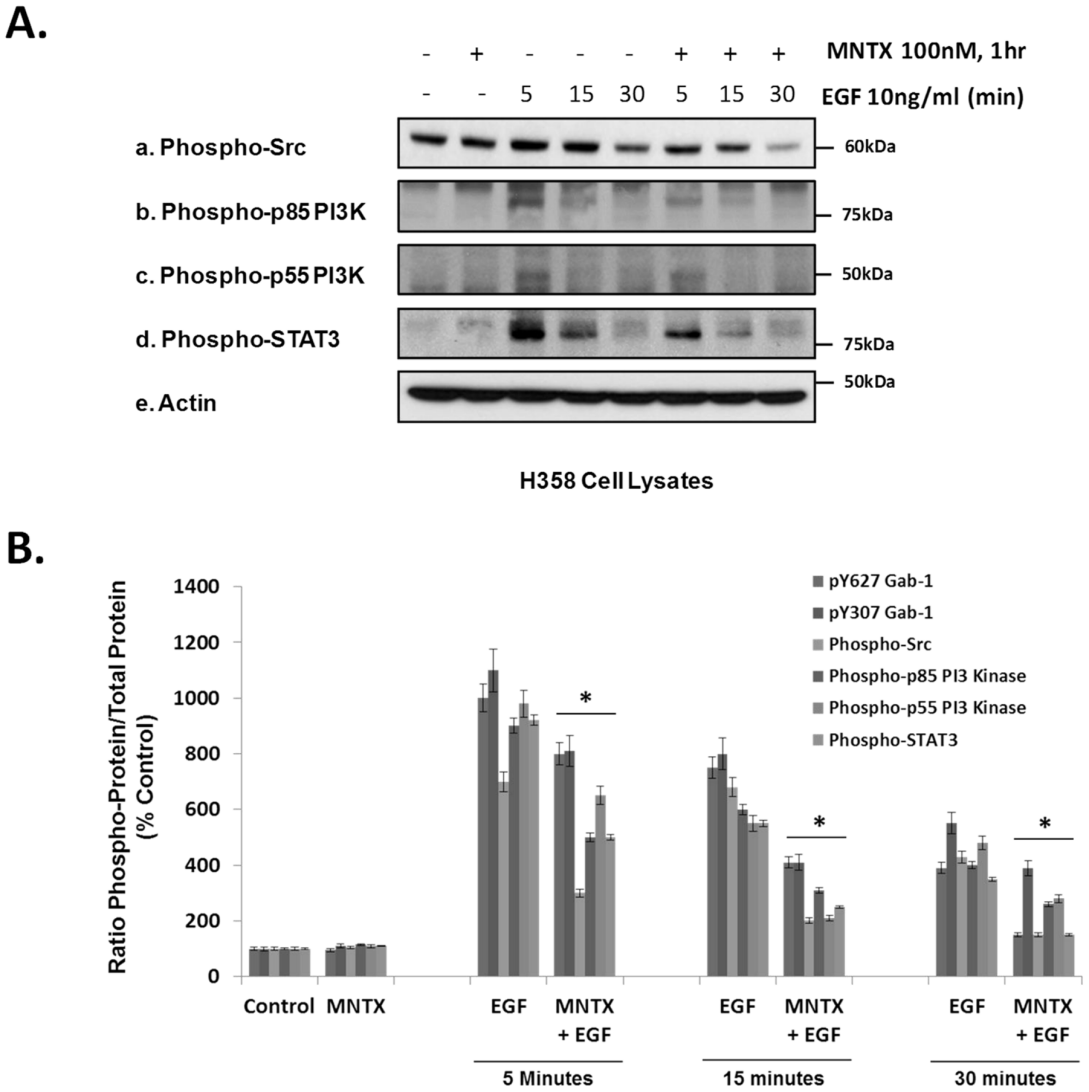
The peripheral mu opioid receptor antagonist, methylnaltrexone (MNTX), inhibits EGF-induced phosphorylation of Src, PI3 kinase and STAT3 in human lung cancer cells. **Panel A**: Human H358 non-small cell lung cancer (NSCLC) cells were either untreated (control) or treated with 100 nM MNTX alone, 10 ng/ml EGF for 5, 15 or 30 minutes, or 100 nM MNTX and 10 ng/ml EGF for 5, 15, or 30 minutes. Cell lysates were obtained and immunoblotted using anti-phospho-Src (pY^416^) (a), anti-phospho-p85/p55 PI3 kinase (pY^458^/pY^199^) (b,c), anti-phospho-STAT3 (pY^705^) (d) and anti-actin (e) antibodies. **Panel B**: Graphical quantitation of immunoreactivity of experiments performed described in Figure 3-B and Panel A with normalization to total specific protein and n = 3 independent experiments per condition. An asterisk (*) indicates a statistically significant difference (p<0.05) from control. The error bars = standard deviation.

Since tyrosine phosphorylation of Gab-1 by various tyrosine kinases including Src promotes binding to signaling molecules including Phosphatidylinositol 3-kinases (PI3Ks) which generate PIP3 and activate downstream effectors including Akt and STAT3 [37], [38], [39], [40], [41], [42], we next examined whether MOR inhibition can also influence Gab-1 binding/effector molecules. Figure 4-A shows that, like Gab-1, EGF challenge of H358 cells induces tyrosine phosphorylation of Src (Tyr416), the regulatory PI3K alpha subunits p85 and p55 (Tyr458/Tyr199), and the transcription factor STAT3 (Tyr705) which peak at ∼5 minutes and are attenuated by MOR inhibition with MNTX in a statistically significant manner (Figure 4-B).

**Figure 5:**
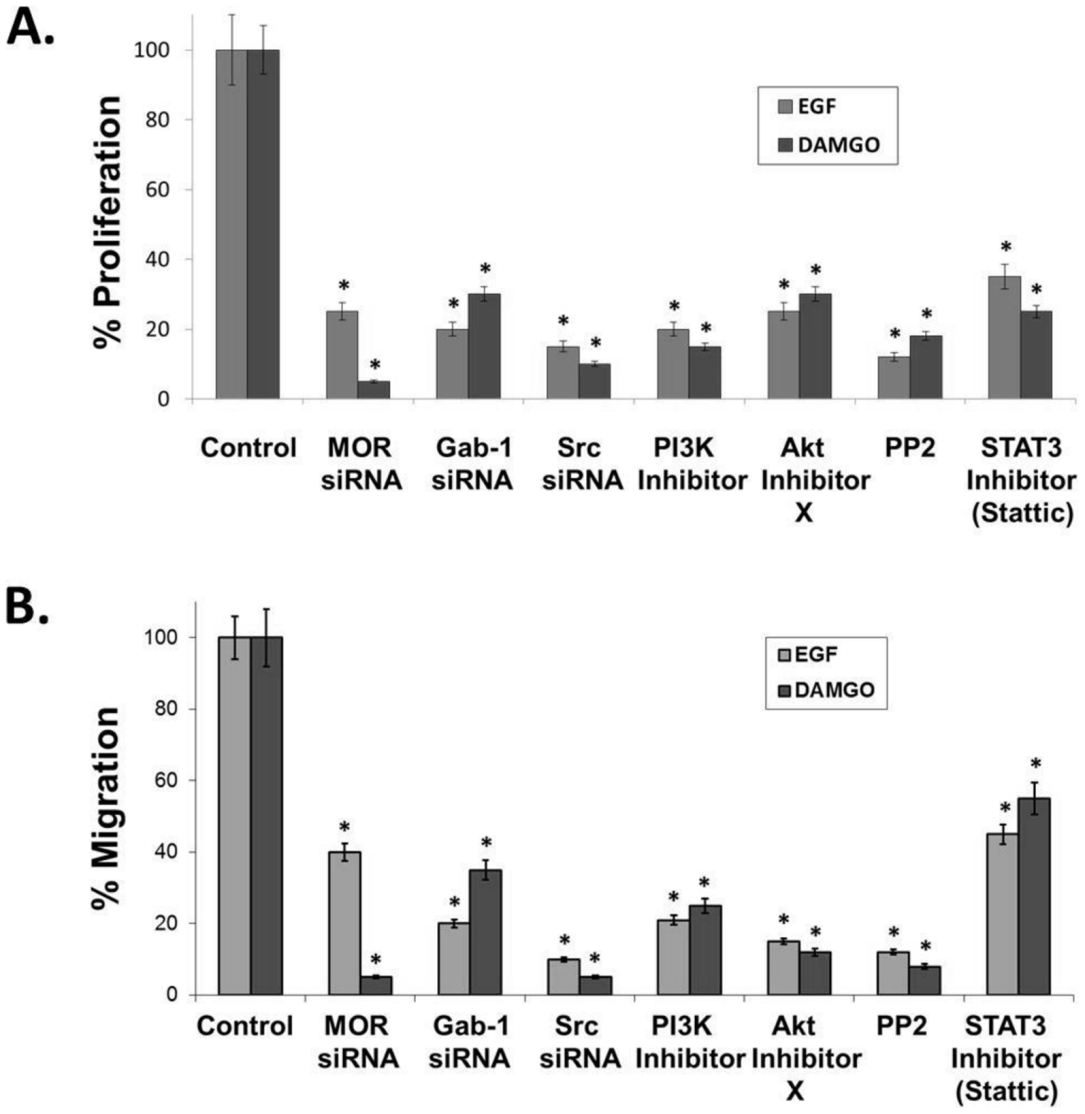
Inhibiting the mu opioid receptor (MOR), Gab-1, Src, PI3 kinase, Akt or STAT3 attenuates EGF and DAMGO-induced proliferation and migration in human lung cancer cells. **Panel A**: Human H358 non-small cell lung cancer (NSCLC) cells were analyzed for EGF and DAMGO-mediated proliferation using a MTS proliferation assay. H358 human NSCLC cells were either untreated (control) or treated with 100 ng/ml epidermal growth factor (EGF) or 100 nM DAMGO for 72 hours with or without pretreatment of cells with the peripheral MOR antagonist, methylnaltrexone (MNTX, 100 nM), MOR siRNA, Gab-1 siRNA, Src siRNA, the PI3 kinase inhibitor LY294002 (10 uM), Akt Inhibitor X (5 uM), the Src family kinase inhibitor PP2 (100 nM) or the STAT3 inhibitor Stattic (10 uM). There is a statistically significant difference (p<0.05, indicated by an asterisks) between control and treatment groups with n = 3 independent experiments per condition and error bars = standard deviation. See the Methods section for experimental details. **Panel B**: Human H358 non-small cell lung cancer (NSCLC) cells were analyzed for EGF and DAMGO-mediated migration using a transwell assay (8 uM pore size). H358 human NSCLC cells were either untreated (control) or treated with 100 ng/ml epidermal growth factor (EGF) or 100 nM DAMGO for 18 hours with or without pretreatment of cells with the peripheral MOR antagonist, methylnaltrexone (MNTX, 100 nM), MOR siRNA, Gab-1 siRNA, Src siRNA, the PI3 kinase inhibitor LY294002 (10 uM), Akt Inhibitor X (5 uM), the Src family kinase inhibitor PP2 (100 nM) or the STAT3 inhibitor Stattic (10 uM). There is a statistically significant difference (p<0.05, indicated by an asterisks) between control and treatment groups with n = 3 independent experiments per condition and error bars = standard deviation. See the Methods section for experimental details.

We next examined the mechanism by which EGF and DAMGO ([D-Ala2, N-MePhe4, Gly-ol]-enkephalin), a synthetic opioid peptide with specificity for MOR) induce proliferation and migration. Figure 5 illustrates that siRNA and/or chemical inhibition of MOR, Gab-1, Src, PI3K, Akt and STAT3 markedly decrease EGF and DAMGO-induced proliferation (Figure 5-A) and migration (Figure 5-B).

**Figure 6:**
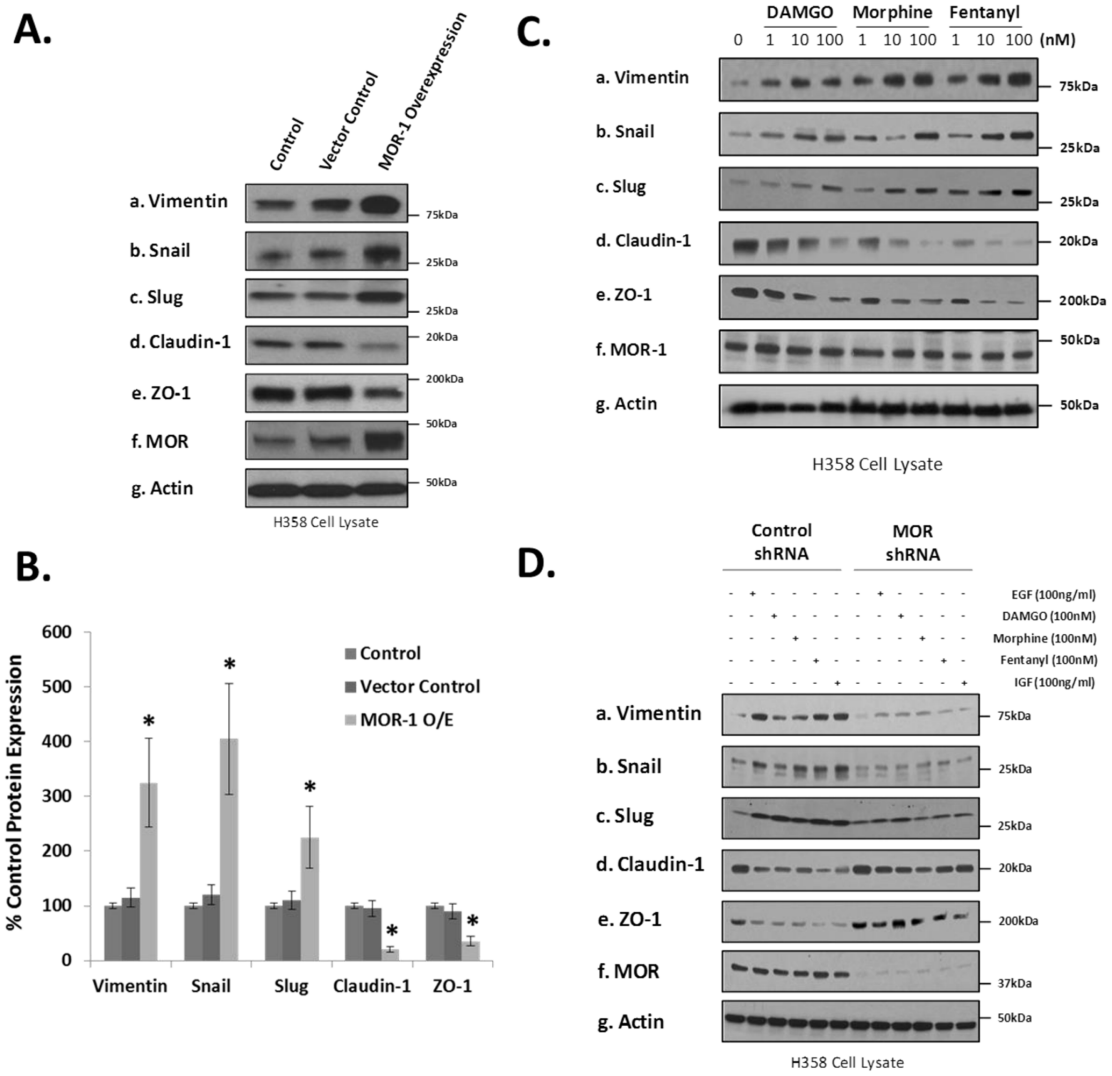
Overexpression or silencing of the mu opioid receptor (MOR) or addition of opioids in human lung cancer cells regulates epithelial mesenchymal transition (EMT). **Panel A**: Control (non-transfected)(C), stable vector control (VC) and MOR1 overexpressing (O/E) H358 cell lines were generated, cell lysates obtained and immunoblotted with EMT markers anti-vimentin (a), anti-Snail (b), anti-Slug (c), anti-claudin-1 (d), anti-ZO-1 (e), anti-MOR (f) and anti-actin (g) antibodies. An increase in vimentin, Snail and Slug expression and a decrease in claudin-1 and ZO-1 expression suggest an epithelial mesenchymal transition. **Panel B**: Graphical quantitation of immunoreactivity of experiments performed described in Panel A with n = 3 independent experiments per condition. An asterisk (*) indicates a statistically significant difference (p<0.05) from control with error bars = standard deviation. **Panel C**: H358 human NSCLC cells were either untreated, treated with 100 ng/ml epidermal growth factor (EGF), 100 nM DAMGO, morphine or fentanyl or 100 ng/ml insulin growth factor (IGF) for 96 hours, cell lysates obtained and immunoblotted with EMT markers anti-vimentin (a), anti-Snail (b), anti-Slug (c), anti-claudin-1 (d), anti-ZO-1 (e), anti-MOR (f) and anti-actin (g) antibodies. A decrease in vimentin, Snail and Slug expression and an increase in claudin-1 and ZO-1 expression suggest inhibition of epithelial mesenchymal transition. **Panel D**: Control shRNA or MOR shRNA H358 cell lines were generated, cell lysates obtained and immunoblotted with EMT markers anti-vimentin (a), anti-Snail (b), anti-Slug (c), anti-claudin-1 (d), anti-ZO-1 (e), anti-MOR (f) and anti-actin (g) antibodies. An increase in vimentin, Snail and Slug expression and a decrease in claudin-1 and ZO-1 expression suggest an epithelial mesenchymal transition.

Since very little is known about MOR regulation of epithelial mesenchymal transformation (EMT) and the molecular mechanisms integrating cancer cell proliferation, migration and EMT, we next examined whether MOR1 overexpression regulates lung cancer EMT. In Figure 6-A,B, we observed that MOR overexpression in human H358 NSCLC cells induced a change in EMT marker expression which is consistent with an epithelial mesenchymal transition [19]. Since the MOR is the main receptor for certain opioids [4], [43], we next evaluated whether opioids can induce EMT in human H358 NSCLC cells. Figure 6-C indicates that DAMGO, morphine and fentanyl all induce EMT in NSCLC in a dose-dependent manner.

**Figure 7:**
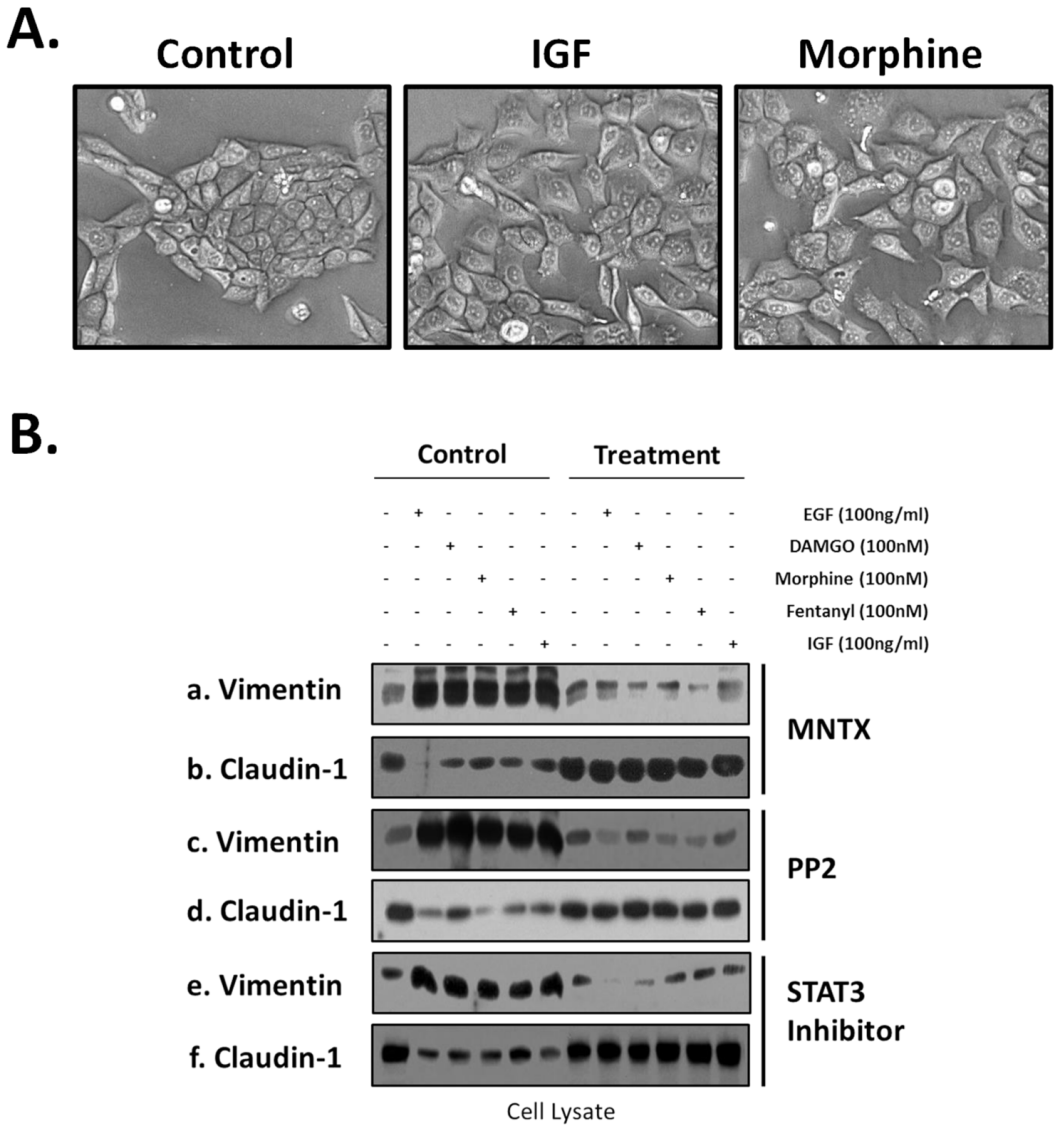
Morphologic and epithelial mesenchymal transition (EMT) marker changes in human lung cancer cells with opioids, growth factors and signal transduction pathway inhibitors. **Panel A**: H358 human NSCLC cells were either untreated (control), treated with 100 nm morphine or 100 ng/ml insulin growth factor (IGF) for 96 hours and brightfield images were obtained (20×). H358 cells grow in colonies with well demarcated borders and strong cell-cell adhesions. In contrast, morphine or IGF-treated H358 cells show a loss of cell-cell adhesions and a change from cuboidal to an elongated phenotype with several cellular projections visible. These changes are consistent with an epithelial mesenchymal transition (EMT). **Panel B**: H358 human NSCLC cells were either untreated, treated with 100 ng/ml epidermal growth factor (EGF), 100 nM DAMGO, morphine or fentanyl or 100 ng/ml insulin growth factor (IGF) for 96 hours with or without pretreatment with the peripheral MOR antagonist, methylnaltrexone (MNTX, 100 nM), the Src family kinase inhibitor PP2 (100 nM) or the STAT3 inhibitor Stattic (10 uM). Cell lysates were then obtained and immunoblotted with EMT markers anti-vimentin (a,c,e) or anti-claudin-1 (b,d,f) antibodies. An increase in vimentin expression and a decrease in claudin-1 expression suggest an epithelial mesenchymal transition.

Considering these H358 human NSCLC cells express basal levels of MOR and respond to opioid treatment, we examined whether silencing (shRNA) of MOR would affect opioid and growth factor-induced EMT. Figure 6-D indicates MOR silencing inhibits opioid and EGF/IGF-induced changes in EMT marker expression. Figure 7-A indicates that H358 cells grow in colonies with well demarcated borders and strong cell-cell adhesions. In contrast, morphine or IGF-treated H358 cells show a loss of cell-cell adhesions and a change from cuboidal to an elongated phenotype with several cellular projections visible. These changes are consistent with an epithelial mesenchymal transition (EMT).

**Figure 8:**
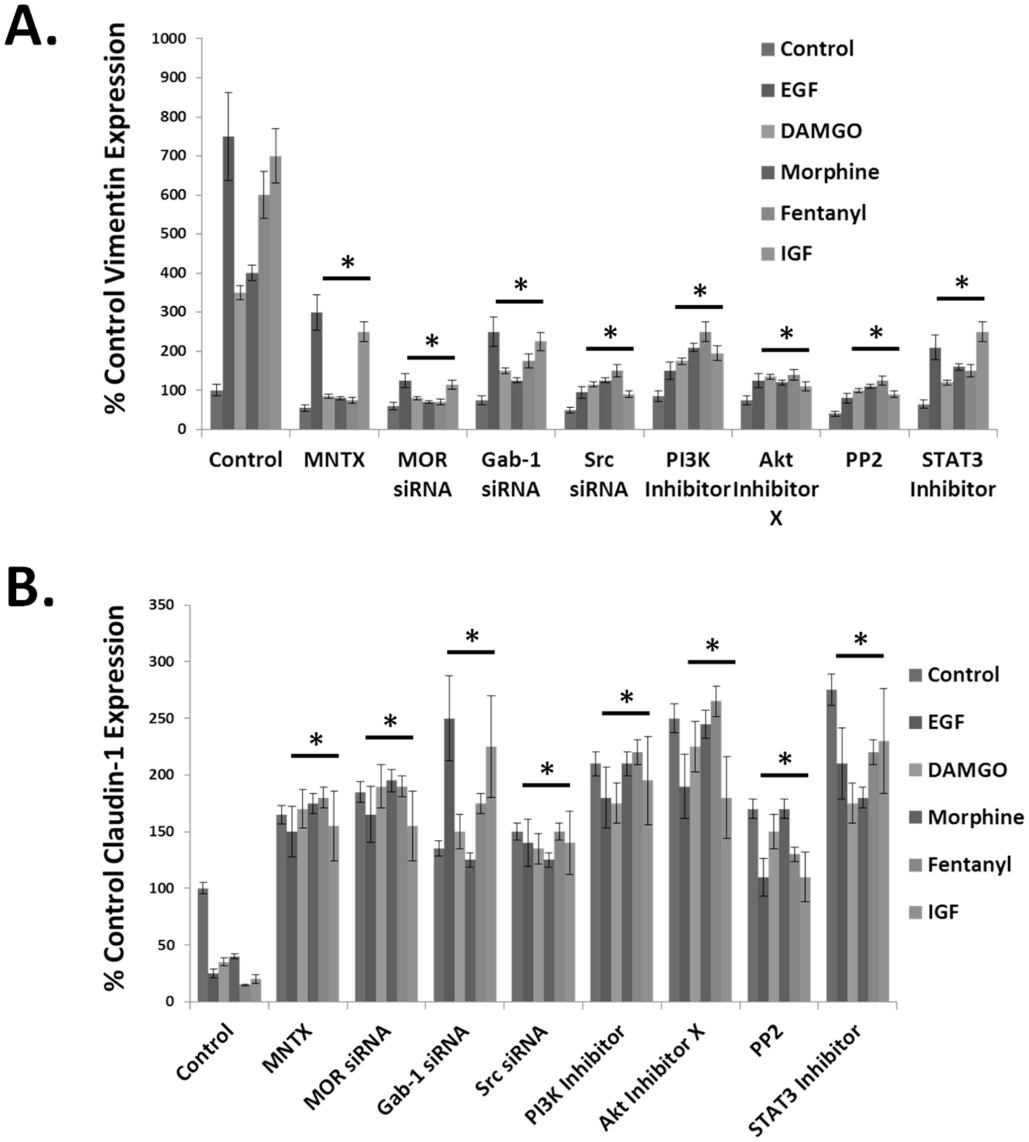
Inhibiting the mu opioid receptor (MOR), Src, Gab-1, PI3 kinase or STAT3 attenuates opioid and growth factor-induced epithelial mesenchymal transition (EMT) in human lung cancer cells. **Panel A**: Graphical representation of the % control vimentin expression. H358 human NSCLC cells were either untreated, treated with 100 ng/ml epidermal growth factor (EGF), 100 nM DAMGO, morphine or fentanyl or 100 ng/ml insulin growth factor (IGF) for 96 hours with or without pretreatment of cells with the peripheral MOR antagonist, methylnaltrexone (MNTX, 100 nM), MOR siRNA, Gab-1 siRNA, Src siRNA, the PI3 kinase inhibitor LY294002 (10 uM), Akt Inhibitor X (5 uM), the Src family kinase inhibitor PP2 (100 nM) or the STAT3 inhibitor Stattic (10 uM). Cell lysates were then obtained and immunoblotted with the EMT marker anti-vimentin antibody. Experiments were repeated in triplicate and immunoreactive bands were analyzed using computer-assisted densitometry. There is a statistically significant difference (p<0.05 indicated by asterisks (*)) between control and treatment groups with error bars = standard deviation. An increase in vimentin expression is suggestive of an epithelial mesenchymal transition. **Panel B**: Graphical representation of the % control claudin-1 expression. H358 human NSCLC cells were either untreated, treated with 100 ng/ml epidermal growth factor (EGF), 100 nM DAMGO, morphine or fentanyl or 100 ng/ml insulin growth factor (IGF) for 96 hours with or without pretreatment of cells with the peripheral MOR antagonist, methylnaltrexone (MNTX, 100 nM), MOR siRNA, Gab-1 siRNA, Src siRNA, the PI3 kinase inhibitor LY294002 (10 uM), Akt Inhibitor X (5 uM), the Src family kinase inhibitor PP2 (100 nM) or the STAT3 inhibitor Stattic (10 uM). Cell lysates were then obtained and immunoblotted with the EMT marker anti-claudin-1 antibody. Three independent experiments per condition were performed and immunoreactive bands were analyzed using computer-assisted densitometry. There is a statistically significant difference (p<0.05 indicated by asterisks (*)) between control and treatment groups with error bars = standard deviation. A decrease in claudin-1 expression is suggestive of an epithelial mesenchymal transition.

We next examined potential signal transduction proteins that could potentially regulate opioid and growth factor-induced EMT. Figure 7-B indicates pretreatment with the peripheral MOR antagonist, methylnaltrexone (MNTX), the Src family kinase inhibitor PP2 or the STAT3 inhibitor Stattic reverse the opioid and growth factor-induced changes in vimentin and claudin-1 expression. In addition, Figure 8 indicates siRNA and/or chemical inhibition of MOR, Gab-1, Src, PI3K, Akt and STAT3 dramatically inhibits EMT (as determined by inhibition of opioid and growth factor-induced increase in vimentin expression and decrease in claudin-1 expression). Both MNTX and the MOR inhibitor naloxone attenuated opioid and growth factor-induced EMT indication a general effect of MOR antagonists on this process (Figures 7 and 8 and supporting data, Figure S1). Taken together, our data suggest MOR plays a central role in the processes of proliferation, migration and epithelial mesenchymal transition alone and in conjunction with opioids and growth factors.

## Discussion

**Figure 9:**
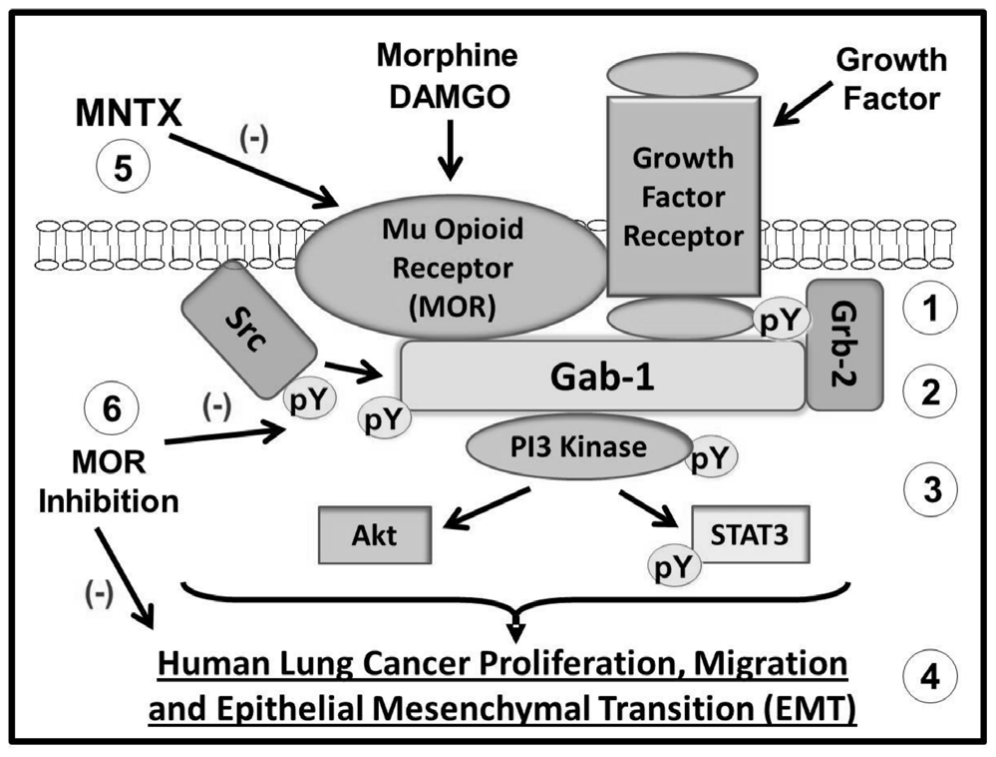
Schematic diagram illustrating mu opioid receptor (MOR) regulation of growth factor receptor signaling and human lung cancer proliferation, migration and epithelial mesenchymal transition (EMT). Activation of growth factor receptors promotes complex formation with the mu opioid receptor (MOR) and Grb-2 as well as Src activation (1). These events induce recruitment of the scaffolding protein, Gab1, to the plasma membrane (2) and consequent recruitment/activation of PI3 kinase, Akt and STAT3 signaling (3) which are required for human lung cancer proliferation, migration and epithelial mesenchymal transition (EMT) (4). Inhibition of MOR (siRNA, shRNA and/or the peripheral MOR antagonist, methylnaltrexone (MNTX)) blocks morphine and DAMGO binding to the mu opioid receptor (5). In addition, inhibiting MOR attenuates growth factor-induced phosphorylation/activation of Src, Gab-1, PI3 kinase, Akt and STAT3 with consequent inhibition of human lung cancer proliferation, migration and EMT (6).

NSCLC, which accounts for ∼80% of all lung cancers, is a disease with high mortality and few treatment options [44], [45]. We have previously reported that the MOR is upregulated in lung tissue from patients with NSCLC [12] and that overexpression of MOR promotes tumor growth and metastasis in human NSCLC xenograft models [13]. Further, data from Fujioka et al., indicate that the MOR regulates EGF-induced signaling events in NSCLC [35]. Considering there is very limited data on opioid regulation of epithelial mesenchymal transition (EMT, a crucial process for cancer progression) and the molecular mechanisms integrating cancer cell proliferation, migration and EMT, this study examined the role of opioids and MOR in these processes [17], [18], [19], [20]. Here we present evidence that activation of growth factor receptors promotes complex formation with the mu opioid receptor (MOR) and Grb-2 as well as Src activation. These events induce recruitment of the scaffolding protein, Gab1, to the plasma membrane and consequent recruitment/activation of PI3 kinase, Akt and STAT3 signaling which are required for human lung cancer proliferation, migration and EMT. Inhibition of MOR (siRNA, shRNA and/or MOR antagonists) blocks morphine and DAMGO binding to the mu opioid receptor. In addition, inhibiting MOR attenuates growth factor-induced phosphorylation/activation of Src, Gab-1, PI3 kinase, Akt and STAT3 with consequent inhibition of human lung cancer proliferation, migration and EMT (see Figure 9).

Our results are consistent with Fujioka et al., who demonstrated that morphine and EGF challenge of human lung cancer cells (H2009, NSCLC) induced Akt and MAPK/ERK activation as well as cell proliferation and invasion [35]. These effects were blocked with the MOR antagonist, naloxone [35]. Further, these authors and others have reported that agonists of MOR can transactivate the EGRF [35], [46], [47], [48]. While the current study did not look at opioid stimulation of EGFR phosphorylation, our results indicate that blocking MOR with the peripheral MOR antagonist MNTX did not affect EGF-induced EGFR tyrosine phosphorylation. Our results further demonstrate that MNTX can block EMT transformation by another growth factor implicated in lung cancer progression, insulin growth factor (IGF) [49], [50], [51]. Whether MOR can regulate IGF receptor phosphorylation/signaling in human NSCLC is currently being investigated in our laboratory.

The current study presents the novel findings that the scaffolding protein, Grb2-associated-binding protein 1 (Gab-1), regulates opioid and growth factor-mediated human lung cancer cell proliferation, migration and EMT transformation. The adaptor protein, Growth factor receptor-bound protein 2 (Grb-2), can directly bind tyrosine phosphorylated sites on the EGRF via its SH2 domain which promotes plasma membrane recruitment of Gab-1 and binding of proline-rich motifs on Gab-1 to SH3 domains on Grb-2 [37]. Further, the proline-rich motifs on Gab-1 can bind to the non-receptor tyrosine kinase Src [37], [52]. We have previously demonstrated that MNTX is a potent inhibitor of Src activation [43], [53]. Our results that MNTX can also inhibit Grb-2/Gab-1 recruitment/activation could explain how MNTX regulates Src activity.

Tyrosine phosphorylation of Gab-1 by Src and other tyrosine kinases promotes recruitment of several signaling molecules including PI3K, PLCgamma, RasGAP and SHP2 [37], [52], [54]. Our current results indicate that MOR antagonism by MNTX blocks activation (tyrosine phosphorylation) of the PI3K regulatory subunits, p85 and p55. Further, inhibition of PI3K activity attenuates opioid- and growth factor-induced human lung cancer cell proliferation, migration and EMT. Whether PLCgamma, RasGAP and/or SHP2 also play a role in MOR regulation of NSCLC progression is currently being investigated in our laboratory.

The transcription factor, Signal transducer and activator of transcription 3 (STAT3), is an important regulator of NSCLC progression [41], [55], [56], [57]. Tyrosine phosphorylation of STAT3 induces dimerization and nuclear translocation [57]. Recently, it has been shown that PI3K can also regulate STAT3 activity [38], [39]. In this study, we have demonstrated that STAT3 regulates opioid and growth factor-induced proliferation, migration and EMT. Further, MNTX inhibits STAT3 tyrosine phosphorylation implying a role for MOR in STAT3 activation. This is in partial agreement with Debruyer et al., 2010, indicating that delta opioid receptor (DOR) agonists can differentially affect migration of HCT-8/E11 human colon cancer cells [58].

Another downstream effector of PI3K is the serine/threonine kinase Akt [42], [59]. Akt contains an SH2 domain that can bind to PIP2 and PIP3 as well as serine, threonine and tyrosine phosphorylation sites [60], [61]. We have previously reported that MNTX inhibits opioid and VEGF-induced Akt activation in human endothelial cells and that overexpression of MOR in human NSCLC activates Akt [4], [13], [62], [63]. In this study, using Akt inhibitor X (a selective inhibitor of Akt phosphorylation and activity), we demonstrate that Akt regulates opioid and EGF-induced human NSCLC cell proliferation, migration and EMT transformation.

EMT is a complex process in which there is a loss of cell-cell adhesion (characterized by a reduction of epithelial cell adhesion proteins including the tight junction proteins, ZO-1 and claudin-1) followed by acquisition of mesenchymal characteristics including a loss of baso-apical polarization, cytoskeletal remodeling and increased cell motility (characterized by increases in specific cytoskeletal proteins (i.e. vimentin) and transcription factors (i.e. Slug and Snail)[16], [17], [18], [19], [20], [22]. We have previously reported that overexpression of MOR1 (the most abundant MOR transcript that consists of exons 1, 2, 3 and 4)[64] in human H358 NSCLC cells induced elongated cellular projections suggestive of a migratory “mesenchymal” phenotype [19]. This prompted us to examine MOR regulation of EMT in human NSCLC cells. While we used a panel of EMT markers, several others exist including Twist, N-cadherin, E-cadherin, ZEB1/2, beta-catenin, fibronectin and desmoplakin [16], [17], [18], [19], [20], [22]. In addition to cancer, EMT has been implicated in several fibrotic diseases by providing a source for myofibroblasts [22], [65]. Whether MOR is involved in the regulation of fibrotic diseases via EMT is currently unknown.

Exogenous opioids are utilized clinically for their analgesic effects, cough and diarrhea suppression and pain relief [66], [67]. Opioid relief of pain is indicated in various conditions such as acute pain after surgery, injury or trauma as well as chronic pain from advanced cancer. Recently, Garcia-Recio et al., 2013, demonstrated that the pain-associated tachykinin, Substance P, and its GPCRs can transactivate HER2/EGFR signaling in human breast cancer cell lines [68]. This study provides a novel mechanism by which pain and inflammation can promote tumor progression. Substance P receptors can heterodimerize with mu opioid receptors and these receptors can transactivate each other [69], [70], [71], [72], [73], [74], [75]. Therefore, it is possible that pain and inflammation could, through Substance P transactivation of MOR, promote EMT in cancer cells. This potential link could be ameliorated with MOR antagonists. Further research is warranted.

Given our previous published data indicating the mu opioid receptor (MOR) is increased in human lung cancer [12], that lung cancer cells do not form visible tumors in MOR knockout mice [12] and that MOR overexpression promotes NSCLC primary tumor growth and metastasis [4], [43], we hypothesized that MOR regulation of EMT might be a plausible explanation for the differences in recurrence rates observed in the epidemiologic studies. Taken together, our data suggests the MOR plays a crucial role in the fundamental cellular EMT changes that occur during lung cancer progression, and provides a plausible explanation for the epidemiologic findings.

## Supporting Information

Figure S1 **The MOR antagonist, naltrexone, inhibits epithelial mesenchymal transition (EMT) in human lung cancer cells.** **Panel A**: H358 human NSCLC cells were either untreated, treated with 100 nM morphine, DAMGO, fentanyl or 100 ng/ml EGF for 96 hours with or without pretreatment of cells with the MOR antagonist, naltrexone (100 nM). Cell lysates were obtained and immunoblotted with EMT markers anti-vimentin (1), anti-Slug (2) and anti-actin (g) antibodies. A decrease in vimentin and Slug expression suggest an inhibition of epithelial mesenchymal transition.
(TIF)
