# Supplementary figures and images for: The Mu Opioid Receptor Promotes Opioid and Growth Factor-Induced Proliferation, Migration and Epithelial Mesenchymal Transition (EMT) in Human Lung Cancer
Source: PLoS One. 2014 Mar 24;9(3):e91577. doi: 10.1371/journal.pone.0091577 (PMC3963855; doi:10.1371/journal.pone.0091577)

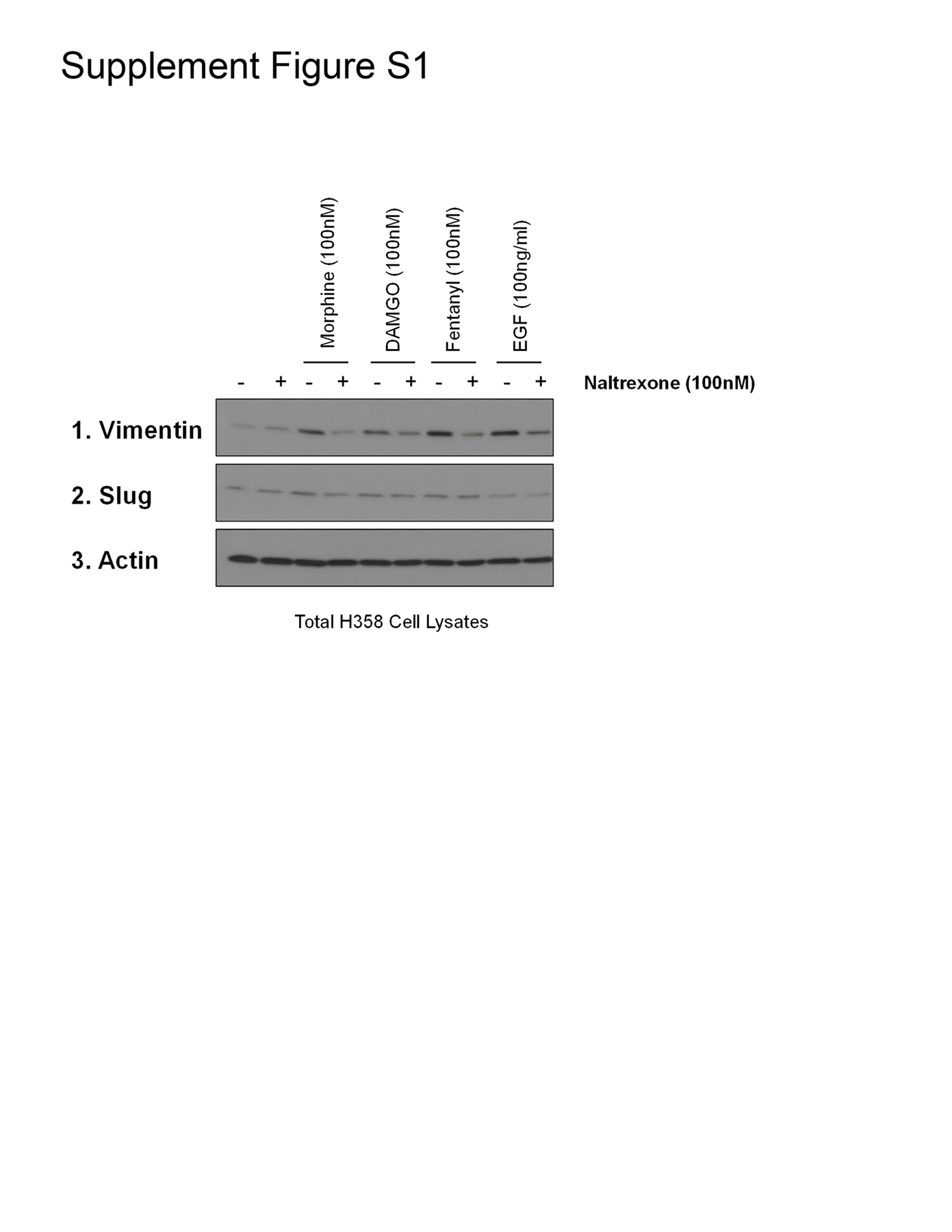

Supplement: Figure S1 — The MOR antagonist, naltrexone, inhibits epithelial mesenchymal transition (EMT) in human lung cancer cells. Panel A: H358 human NSCLC cells were either untreated, treated with 100 nM morphine, DAMGO, fentanyl or 100 ng/ml EGF for 96 hours with or without pretreatment of cells with the MOR antagonist, naltrexone (100 nM). Cell lysates were obtained and immunoblotted with EMT markers anti-vimentin (1), anti-Slug (2) and anti-actin (g) antibodies. A decrease in vimentin and Slug expression suggest an inhibition of epithelial mesenchymal transition. (TIF) [file pone.0091577.s001.tif]
